# Quantitative microbial risk assessment of extended-spectrum β-lactamase-producing *Escherichia coli* transfer from broiler litter to fresh lettuce consumption

**DOI:** 10.1016/j.soh.2026.100152

**Published:** 2026-02-19

**Authors:** Nunzio Sarnino, Subhasish Basak, Lucie Collineau, Roswitha Merle

**Affiliations:** a Freie Universität Berlin, Institute of Veterinary Epidemiology and Biostatistics, Veterinary Centre for Resistance Research Berlin, Germany; b University of Lyon - French Agency for Food, Environmental and Occupational Health and Safety (ANSES), Epidemiology and Surveillance Support Unit, Lyon, France

**Keywords:** Extended-spectrum β-lactamase-producing *Escherichia coli*, Environmental transmission pathways, Human exposure assessment, Broiler production, Quantitative microbial risk assessment

## Abstract

**Background:**

Extended-spectrum β-lactamase-producing *Escherichia coli* (ESBL *E. coli*) from broiler chicken production pose potential public health risks via multiple environmental and foodborne pathways. We developed a modular quantitative microbial risk assessment (QMRA) model linking four components, namely farm, soil, river, and lettuce consumption, to predict human environmental exposure to ESBL *E. coli* originating from broiler flocks.

**Methods:**

A stochastic farm module simulated broiler colonization over a 36-day cycle and generated end-cycle litter loads. Field modules represented first-order decay, partitioning, and runoff to rivers; irrigation transfer yielded lettuce contamination for a 100 g serving. We estimated exposure, mapped gastrointestinal colonization to urinary tract infection (UTI) via conditional probabilities, and expressed the burden as disability-adjusted life years (DALYs) per serving. Global sensitivity analyses identified main exposure drivers. Environmental time was indexed as days since litter application and the planting interval denoted days from litter application to planting.

**Results:**

The farm model produced mean end-cycle litter of 1.6 × 10^4^ CFU/g and near-complete flock colonization within one week. Soil surface loads declined from 3.2 × 10^7^ CFU/m^2^ to 8.6 × 10^5^ CFU/m^2^ by day 100. Runoff yielded river concentrations of 6.0 × 10^−2^ CFU/mL after 10 days. Exposure from lettuce consumption ranged from 1.7 CFU/100 g to 7.6 × 10^−3^ CFU/100 g; simple household washing cut exposure by ∼90 %. Global sensitivity analysis identified soil-water partitioning and decay rates as the most important parameters of exposure variability. For health endpoints, UTI risk per serving ranged from 4.6 × 10^−12^ to 9.0 × 10^−9^, and DALY per serving ranged between 10^−10^ and 10^−8^.

**Conclusions:**

Predicted health burdens decreased markedly with consumer washing and longer intervals between litter application and lettuce planting. Residual contamination persists, indicating value in evaluating the effectiveness of manure treatments and irrigation-water quality interventions on reducing environmental loads and human risk.

## Highlights

• Developed an integrated, modular quantitative microbial risk assessment (QMRA) model tracing extended-spectrum β-lactamase (ESBL)-producing Escherichia coli from broiler farm to lettuce consumption.
• Estimated that standard household washing reduces exposure by ∼90%.
• Predicted that extending the interval between litter application and planting can reduce human exposure.
• Identified soil-water partitioning and environmental decay as dominant drivers of exposure variability.
• Quantified health burden from environmental transfer of ESBL-producing E. coli using urinary tract infection (UTI) risk and disability-adjusted life years (DALYs).

## 1 Introduction

Antimicrobial resistance (AMR) is steadily reducing the effectiveness of antibiotics in human and veterinary medicine. In broiler farming, antimicrobial use may create strong selective pressure and accelerate the emergence and spread of resistant bacteria [1]. A prime example is extended-spectrum β-lactamase-producing *Escherichia coli* (ESBL *E. coli*), which can break down β-lactam antibiotics. These organisms pose a serious public health threat and could increase healthcare costs [[2], [3], [4]].

Resistant strains may enter broiler houses in multiple ways. Day-old chicks may arrive already colonized [5], or resistance may pass vertically from breeder flocks [[6], [7], [8]]. Environmental contamination and biosecurity lapses offer additional routes [9,10]. Although the prevalence of resistance at chick placement is generally low [11], within-flock spread frequently leads to near complete colonization within a few days [12].

Once broiler houses are emptied, litter used as fertilizer potentially serves as a reservoir of ESBL *E. coli.* These bacteria can survive in soil for extended periods [13], enter surface and groundwater through runoff [14], and contaminate produce [15,16]. Humans may then be exposed via contaminated food, recreational water contact, or other environmental pathways [[17], [18], [19], [20]]. Environmental factors, notably rainfall events, can significantly influence bacterial concentrations in rivers, often leading to transient peaks [21,22]. Furthermore, the proximity to animal production sites seems to be an additional risk factor [20,23].

Litter management may reduce ESBL *E. coli* loads. Thermophilic composting inactivates pathogens and lowers bacterial counts [24,25]. Respecting pre-harvest intervals limits the environmental spread [26]. Advanced composting and short-term litter storage further reduce resistant bacterial populations [[27], [28], [29], [30]]. However, bacterial survival depends on environmental conditions, underscoring the need for site-specific risk assessments and tailored interventions [13,31,32].

The interval between manure application and crop planting or harvest is a practical scheduling variable for growers [33]. During this interval, manure-derived bacteria undergo environmental decay in soil that can change subsequent contamination potential [34].

To our knowledge, the specific influence of this interval on harvest contamination has not been quantified within an integrated quantitative microbial risk assessment (QMRA) model, motivating its inclusion in our objectives.

QMRA frameworks offer a systematic approach to characterize the propagation of resistant pathogens and to evaluate the potential effect of control measures [[35], [36], [37]].

Within the ENVIRE project (https://www.envire-project.de/), we developed an integrated QMRA model to simulate how ESBL *E. coli* move from broiler farms under European conditions into the environment and reach humans. Our objectives were to:

1. Predict ESBL *E. coli* loads in raw broiler litter at the end of a conventional production cycle;
2. Characterize transmission pathways from litter to soil, water, and crops;
3. Identify the dominant drivers of exposure at consumption;
4. Explore how the time between litter application and planting shapes contamination at harvest;
5. Estimate human exposure and disability-adjusted life years (DALYs) lost via lettuce consumption.

Other potential routes (for example direct crop contamination from soil) were not included because our previous work [32] found that crop contamination primarily occurs via irrigation water.

This study is, to our knowledge, the first modular QMRA to connect broiler farm, amended soil, receiving water, lettuce consumption for ESBL *E. coli*, using one internally consistent set of assumptions across modules. We (1) link flock colonization dynamics to end-cycle litter loads; (2) propagate soil partitioning and hydrological wash-off to river concentrations; (3) map irrigation transfer to edible portions; and (4) quantify health burden (DALYs). This integrated framing extends prior single-pathway models and generic *E. coli* assessments by targeting ESBL strains and explicitly testing time-since-application scenarios relevant to produce safety.

## 2 Materials and methods

### 2.1 Model description

**Fig. 1:**
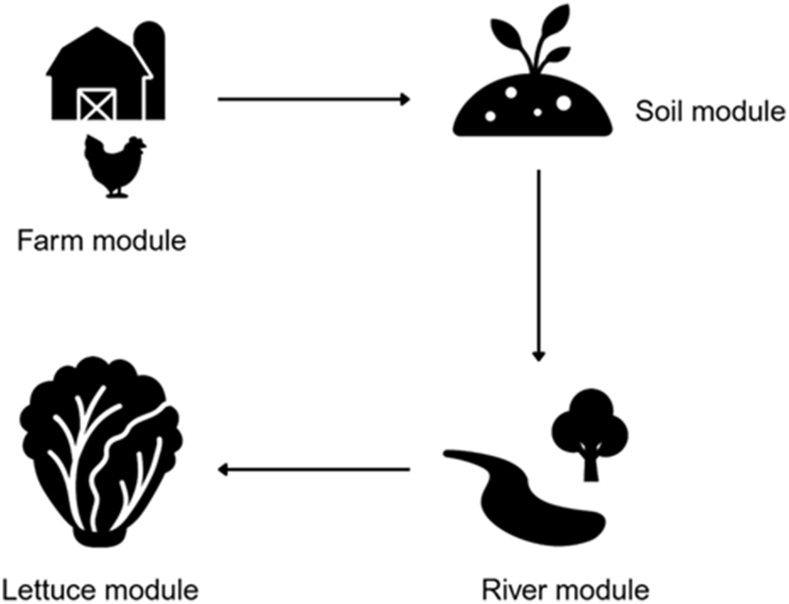
Diagram of the quantitative microbial risk assessment (QMRA) model linking the farm, soil, river, and lettuce modules.

We built a modular QMRA model to estimate human exposure to ESBL *E. coli* from broiler production. The model links four modules—farm, soil, river, and lettuce consumption—to trace contamination from its source all the way to human exposure (Fig. 1).

**Fig. 2:**
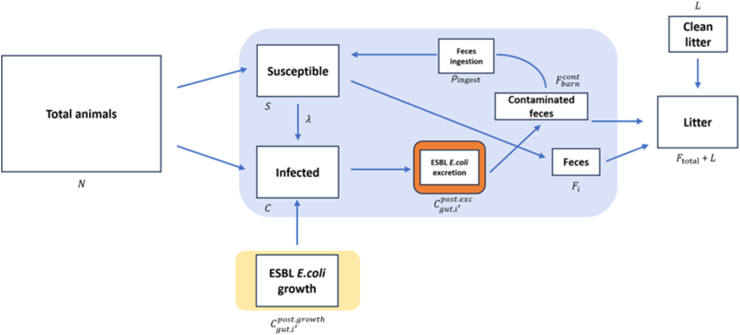
Diagram of the farm module simulating the transmission dynamics of extended-spectrum β-lactamase (ESBL)-producing *Escherichia coli* within a broiler flock.

In the farm module, we simulated a 36-day broiler cycle with a stochastic susceptible-infectious compartmental model. Broilers enter as susceptible (S) or colonized (C) at a set prevalence ($p_{\text{init}}$) (Table 1). Gut bacterial growth (r) follows a logistic curve; infection may spread via ingestion ($\rho_{\text{ingest}}$) of contaminated feces ($F_{barn}^{cont}$). Each day, the model updates broiler age, infection duration, fecal output, and bacterial shedding. ESBL *E. coli* levels accumulate in the barn litter and then decline by natural decay (Fig. 2).

**Table 1:** Key input variables for the farm module.

Variable	Description	Unit	Value	Source
α	Proportion of ingested water lost by metabolism	Proportion	0.7	[39]
*ε*	Excretion rate of ESBL *Escherichia coli*	Fraction	0.3	[39]
$D_{\max}$	Total duration of the fattening period	d	36	Assumed
$r_{\min}$	Minimum growth rate for ESBL *E. coli* in the broiler's intestine	log_10_(CFU/d)	0	[39]
$r_{\max}$	Maximum growth rate for ESBL *E. coli* in the broiler's intestine	log_10_(CFU/d)	5	[39]
K	Maximum amount of bacteria in the substrate (the intestinal content in this case)	CFU/g	10^6^	[12]
ϕ	Environmental decay rate of ESBL *E. coli* in broiler litter	%	0.5	[39]
$\rho_{\text{ingest}}$	Proportion of ingested contaminated feces along with feed	Fraction	0.014	[39]
β	Transmission rate	CFU/day	0.31	[40]
$w_{\min}\left(d\right)$	Minimum water consumption on day *d*	g	21.16–10.35	[41]
$w_{\max}\left(d\right)$	Maximum water consumption on day *d*	g	42.66–31.18	[41]
g(d)	Broiler's average daily gain in weight on day *d*	g	21–100	[42]
I(d)	Broilers average feed intake on day *d*	g	20–189	[42]
A	Size of the farm	m^2^	100	Assumed
$\rho_{\text{farm}}$	Stocking density	kg/m^2^	39	User defined
$w_{\text{tar}}$	Weight of the broilers at the end of the fattening period (36th day)	kg	2.332	[39]
$c_{\text{init}}$	Total ESBL *E. coli* burden in colonized one day old chicks	CFU	100	[39]
$p_{\text{init}}$	Initial prevalence of colonized broilers	Fraction	0.01	Expert opinion, Robé
L	Litter quantity per square meter	g/m^2^	1000	[39]

Abbreviations: ESBL, extended-spectrum β-lactamase; CFU, colony-forming unit.

Throughout this article, we use the term “litter” to refer to the mixture of broiler manure, bedding, feathers, and other materials commonly removed at the end of a production cycle, while “clean litter” is the fresh bedding material added at the start of a production cycle.

The soil module applies broiler litter to an agricultural field—assuming zero delay between chicken harvest and litter spread—and models ESBL *E. coli* decay using an exponential rate fitted to experimental data from Sharma et al. [34]. While poultry litter is often treated, this is not a universal or legally mandated practice across the European Union (EU). There are no specific regulations requiring all poultry litter to be treated or specifying an interval between removal from the barn and field application. Therefore, in our model, we consider a worst-case scenario in which broiler litter is applied to fields immediately after removal from poultry houses.

The river module estimates daily bacterial runoff from the field to the river using a simplified soil and water assessment tool (SWAT)-inspired approach [38]. We considered water temperature, depth, solar radiation, and salinity, then applied wash-off fractions and in-water decay to predict river concentrations.

The lettuce module simulates multiple planting events over a growing season. On irrigation days, bacteria adhere to leaves in proportion to river concentration and retention volume. From irrigation to harvest, loads decrease according to a biphasic decay model, and post-harvest washing further reduces bacterial counts. Exposure is reported as colony-forming units (CFUs) per 100 g serving. In this module, we assume that the lettuce is consumed on harvest day.

Across all modules, we employed a stochastic approach; each module ran 1000 simulations to reflect natural variability in environmental conditions and individual behavior. For each result, we defined the standard deviation (SD) as descriptive measure of dispersion around the mean and the 95 % uncertainty interval (*UI*) as the 2.5th and 97.5th percentiles of the resulting empirical distribution.

#### 2.1.1 Model credibility

We harmonized units and variable definitions across modules, constrained inputs to ranges reported in peer-reviewed studies or agronomic practice, and ran all analyses from scripted, version-controlled code. To support external validity, we planned piecewise checks against independent evidence, comparing our results with experimental studies and other QMRA.

### 2.2 Farm module

The farm module simulates the transmission dynamics of ESBL *E. coli* between two primary reservoirs: the broilers’ gastrointestinal tract and the litter within the barn environment. A single simulation of this stochastic module corresponds to a complete broiler production cycle of $D_{\max}$ days of a single flock of broilers. The module operates within a discrete-time framework with a daily time step. For each day $d=1,2,\ldots ,D_{\max}$ of the production cycle, a series of barn-level events, each representing distinct mechanisms in the transmission dynamics of ESBL *E. coli* within the barn environment, is executed once per day. These events form the core components of the farm module and are detailed following the model initialization.

#### 2.2.1 Module initialization

The farm module is initialized using the total number of broilers in a flock computed as,

(1) $$\begin{array}{c} N=\left(\frac{\rho_{\text{farm}}}{w_{\text{tar}}}\times A\right), \end{array}$$

where $\rho_{\text{farm}}$ is the farm stocking density (kg/m^2^), $w_{\text{tar}}$ is the target weight (kg) at day $D_{\max}$, and A is the farm area (m^2^). Further, let $N_{\text{col}}\left(d\right)$, for $d=1,2,\ldots ,D_{\max}$, denote the total number of colonized broilers in the flock on day d, with an initial proportion $p_{\text{init}}$ used to set $N_{\text{col}}\left(1\right)$. The flock is partitioned into two compartments: susceptible (S) and colonized (C), with no recovery assumed during the production cycle; their respective initial sizes are $\left(N-N_{\text{col}}\left(1\right)\right)$ and $N_{\text{col}}\left(1\right)$.

For i∈C∪S and $d=1,2,\ldots ,D_{\max}$, let $C_{gut,i}^{init}\left(d\right)$ denote the initial total CFU counts of ESBL *E. coli* present in the i-th broiler's gut on day d. $C_{gut,i}^{init}\left(1\right)$ is set to 0 CFU for every i∈S, and $C_{gut,i^{\prime }}^{init}\left(1\right)$ is set to $c_{\text{init}}$ (in CFU), for every $i^{\prime }\in C$. Moreover, on day 1, the barn litter is assumed to be completely free of ESBL *E. coli*, with no CFUs present. Consequently, the value of both $C_{barn}^{init}\left(1\right)$ and $F_{barn}^{cont}\left(1\right)$, representing respectively the total initial CFU count and amount of contaminated feces in the litter on day 1, are set to zero.

#### 2.2.2 Feces excretion

The first barn-level event implemented by the farm module is the production of feces by the broilers. On day d, for $d=1,2,\ldots ,D_{\max}$, the i-th broiler's feces output denoted by $F_{i}\left(d\right)$, for i∈C∪S, is calculated as,

(2) $$\begin{array}{c} F_{i}\left(d\right)=w\left(d\right)\cdot \alpha +I\left(d\right)-g\left(d\right)\;\text{.} \end{array}$$

Here, w(d) is a uniformly distributed random variable with limits $w_{\min}\left(d\right)$ and $w_{\max}\left(d\right)$, representing respectively the day-specific bounds on water consumption of broilers. α is a fractional reduction factor for water. I(d) and g(d) respectively denote the average feed intake (in kg/d) and daily weight gain (in kg/d) of a broiler Ross 308. After the excretion of day, the total amount of contaminated feces in the barn environment until the beginning of day (d+1) is given by,

(3) $$\begin{array}{c} F_{barn}^{cont}\left(d+1\right)=F_{barn}^{cont}\left(d\right)+\sum_{i^{\prime }\in C,}^{N_{\text{col}}\left(d\right)}F_{i^{\prime }}\left(d\right)\text{.} \end{array}$$

#### 2.2.3 ESBL *E. coli* excretion

The event ESBL *E. coli* excretion models the excretion of ESBL *E. coli* in the barn environment along with the feces by the colonized broilers. The CFU counts of ESBL *E. coli* remaining in the $i^{\prime }$-th colonized broiler's gut, for $i^{\prime }\in C$, on day d after excretion, is given by,

(4) $$\begin{array}{c} C_{gut,i^{\prime }}^{post.exc}\left(d\right)=C_{gut,i^{\prime }}^{init}\left(d\right)\times \left(1-\varepsilon \right)\text{,} \end{array}$$

where ϵ is the excretion rate. The total cumulated load of ESBL *E. coli* (in CFU) in the litter after excretion on day d, for $d=1,2,\ldots ,D_{\max}$, is obtained as,

(5) $$\begin{array}{c} C_{barn}^{post.exc}\left(d\right)=C_{barn}^{init}\left(d\right)+\sum_{i^{\prime }\in C}^{N_{\text{col}}\left(d\right)}C_{gut,i^{\prime }}^{init}\left(d\right)\times \varepsilon \;\text{.} \end{array}$$

#### 2.2.4 ESBL *E. coli* growth inside broilers’ guts

Within colonized broilers, bacterial growth event in the gut is modeled with a logistic growth function [43]. With K being the carrying capacity and the growth rate $r_{i^{\prime }}∼U\left(r_{\min},r_{\max}\right)$ distributed uniformly, the CFU counts of ESBL *E. coli* in the gut of $i^{\prime }$-th colonized broiler, for $i^{\prime }\in C$, on day d after the growth event is given by,

(6) $$\begin{array}{c} C_{gut,i^{'}}^{post.growth}\left(d\right)=\frac{K}{1+\left[\frac{K-C_{gut,i^{'}}^{post.exc}\left(d\right)}{C_{gut,i^{'}}^{post.exc}\left(d\right)}\right]\exp\left(-r_{i^{'}}\right)}\; \end{array}.$$

This formulation captures saturation effects and within-flock variability through random draws for  $r_{i^{\prime }}$.

#### 2.2.5 Feces ingestion

The next barn-level event is the ingestion of feces where each broiler is assumed to accidentally ingest a certain proportion $\rho_{\text{ingest}}$ of contaminated fecal material from the barn environment, along with their daily feed, using the same approach as Becker et al. [39]. This triggers the colonization of susceptible, non-colonized broilers, while the already colonized ones remain persistently colonized. The total amount of contaminated feces (in g) accidentally ingested by the i-th broiler, for i∈C∪S, on day d is given by,

(7) $$\begin{array}{c} F_{ingest,i}^{cont}\left(d\right)=I\left(d\right)\rho_{\text{ingest}}\;\text{.} \end{array}$$

Let $N_{\text{new}}\left(d\right)$ denote the expected number of newly colonized broilers on day d through accidental ingestion of contaminated feces. At this stage, the farm module converts the same number of susceptible broilers to the colonized state by simple random sampling without replacement. Simultaneously, the flock is re-partitioned into three mutually exclusive and exhaustive compartments, namely, susceptible (S), newly colonized ($C^{*}$) on day d, and previously colonized (C), with respective sizes $\left(N-N_{\text{col}}\left(d\right)-N_{\text{new}}\left(d\right)\right)$, $N_{\text{new}}\left(d\right)$ and $N_{\text{col}}\left(d\right)$. The quantity of ESBL *E. coli* ingested by already-colonized broilers is considered negligible compared with their gut load and is therefore neglected. The gut concentrations for broilers belonging to all the three compartments are updated as,

(8) $$\begin{array}{c} C_{gut,i}^{init}\left(d+1\right)=\left\{ \begin{array}{c} F_{ingest,i}^{cont}\left(d\right)\;\frac{C_{barn}^{post.exc}\left(d\right)}{F_{barn}^{cont}\left(d+1\right)},\text{for}\;i\in C^{*},\\ C_{gut,i}^{post.growth}\left(d\right),\text{for}\;i\in C,\\ C_{gut,i}^{init}\left(d\right),\text{for}\;i\in S.\; \end{array}\right.\; \end{array}$$

Simultaneously the ESBL *E. coli* load in the barn environment is also updated as,

(9) $$\begin{array}{c} C_{barn}^{post.ingest}\left(d\right)=C_{barn}^{post.exc}\left(d\right)-\sum_{\;i^{\prime \prime }\in C^{*}}^{N_{\text{new}}\left(d\right)}F_{ingest,i^{\prime \prime }}^{cont}\left(d\right)\frac{C_{barn}^{post.exc}\left(d\right)}{F_{barn}^{cont}\left(d+1\right)}\;\text{.} \end{array}$$

However, the quantity of contaminated feces ingested from the barn environment by all the broilers is considered negligible compared to $F_{barn}^{cont}\left(d+1\right)$, and hence it is kept unchanged.

#### 2.2.6 Transmission

The transmission event is used to estimate $N_{\text{new}}\left(d\right)$. The framework proposed by Dame-Korevaar [40] is adapted as a conceptual basis but extended to honor flock heterogeneity and mass balance constraints. The module is based on the following assumptions: (1) broilers acquire ESBL *E. coli* only by accidentally ingesting contaminated litter and broiler-to-broiler transmission through direct contact is ignored; (2) the simulation operates on a fixed time-step of one day, so each broiler experiences at most one exposure decision per day. The force of infection λ(d) relates to the environmental concentration of ESBL *E. coli* to $N_{\text{new}}\left(d\right)$, and is derived as,

(10) $$\begin{array}{c} \lambda =\beta \times {l\text{og}}_{10}\left[\frac{C_{barn}^{post.exc}\left(d\right)}{F_{barn}^{cont}\left(d+1\right)}\right]\text{,} \end{array}$$

where β is the transmission coefficient. The expected number of new colonization during the interval on day d is obtained as,

(11) $$\begin{array}{c} N_{\text{new}}\left(d\right)=\left[N-N_{\text{col}}\left(d\right)\right]\left\{1-e\text{xp}\left[-\lambda \left(d\right)\right]\right\}\;\text{.} \end{array}$$

On day (d+1), the two compartments $C^{*}$ and C are merged as C and the corresponding size is updated as $N_{\text{col}}\left(d+1\right)=N_{\text{col}}\left(d\right)+N_{\text{new}}\left(d\right)\text{.}$ This ensures that the newly colonized broilers contribute to excretion of contaminated feces and experience growth of ESBL *E.coli* inside their guts.

#### 2.2.7 Environmental decay

The final barn-level event is the environmental decay which models the reduction in ESBL *E. coli* population in the barn environment due to absence of suitable growth conditions and thus preventing indefinite bacterial build-up. For a decay rate  ϕ, the initial barn load for the next day after environmental decay, is obtained as,

(12) $$\begin{array}{c} C_{barn}^{init}\left(d+1\right)=C_{barn}^{post.ingest}\left(d\right)\times \left(1-\phi \right)\;.\; \end{array}$$

#### 2.2.8 Final concentration calculation

At the end of a single simulation of the farm module on day $D_{\max}$, the final ESBL *E. coli* concentration in litter (in CFU/g) denoted by  $C_{\text{final}}$, is obtained by dividing the total environmental load by the sum of accumulated feces and the mass of fresh bedding L,

(13) $$\begin{array}{c} C_{\text{final}}=\frac{C_{barn}^{init}\left(D_{\max}+1\right)}{F_{\text{total}}+L}\text{,} \end{array}$$

where $F_{\text{total}}=\sum_{d=1}^{D_{\max}}\sum_{i\in C\cup S}^{N}F_{i}\left(d\right)$ is the aggregated feces mass by day $D_{\max}$.

### 2.3 Soil module

All parameter values and distributional assumptions for the soil and river modules can be found in Table 2.

**Table 2:** Key input variables for the soil, river, exposure, and lettuce-exposure modules.

Variable	Description	Unit	Value	Source
$\lambda_{s}$	First-order die-off rate of ESBL *Escherichia coli* in litter-amended top-soil	d^−1^	0.0362	[34]
$R_{\text{app}}$	Broiler litter applied to field surface	kg/m^2^	2	Assumed
$K_{d}$	Bacteria partition coefficient	Fraction	0.95	[14]
$f_{\text{wash}}$	Mobile-phase cells washed off per run-off event	Fraction	0.50	[14]
T	River-water temperature	°C	21–28	[18]
EC	Salinity	Fraction	0.035–0.075	[18]
IA	Global solar irradiance	ly/h	17.3–25.4	[18]
e	Light–extinction coefficient	m	0.26–0.31	[18]
H	Mean water-column depth	m	0.5–6.0	[18]
V	Effective pool volume at water site	L	6.75 × 10^7^–8.25 × 10^7^	[18]

Abbreviation: ESBL, extended-spectrum β-lactamase.

#### 2.3.1 *E. coli* decay in soil

Empirical data on *E. coli* decay in litter-amended soil were obtained from Sharma et al. [34], measured at several days post-inoculation.

To characterize *E. coli* decline, an exponential decay function was assumed to follow:

(14) $$\begin{array}{c} y=C\times \exp\left(-\lambda_{s}\times t\right)\text{,} \end{array}$$

where *y* is the *E. coli* concentration at time *t* (in d), *C* is the initial concentration parameter, and $\lambda_{s}$ is the decay rate constant. The parameters *C* and $\lambda_{s}$ were estimated via nonlinear least squares:

(15) $$\begin{array}{c} \underset{C,\lambda_{s}}{\arg\;\;\min}\sum_{i=1}^{n}{\left[y_{i}-C\times \exp\left(-\lambda_{s}\times t_{i}\right)\right]}^{2}\text{,} \end{array}$$

#### 2.3.2 Litter application

The temporal evolution of the ESBL *E. coli* load on the soil was modeled for each Monte Carlo simulation *i* by a first-order decay of the initial litter-derived load. In our model, we therefore describe:

(16) $$\begin{array}{c} L_{i}\left(t\right)=C_{\text{man},i}\times R_{\text{app}}\times 1000\times \exp\left(-\lambda_{s}\;t\right)\text{,} \end{array}$$

where $L_{i}\left(t\right)$ (in CFU/m^2^) is the ESBL *E. coli* load at time t (days) after application, $C_{\text{man},i}$ (in CFU/g) is the Monte Carlo draw of the litter concentration from the farm module output $C_{\text{final}}$, $R_{\text{app}}$ (in kg/m^2^) is the application rate, the factor 1000 converts kilograms to grams, and $\lambda_{s}$ is the decay constant.

### 2.4 River transport and decay model

We route the soil-derived load $L_{i}\left(t\right)$ into a simplified runoff and in-stream decay scheme for each Monte Carlo iteration i and day t. Surface wash-off is computed once per day based on a constant fraction of freely mobile bacteria; this wash-off is then instantly delivered to the receiving water body (i.e. travel time is assumed negligible on the daily time-step).

#### 2.4.1 Wash-off load

(17) $$\begin{array}{c} W_{i}\left(t\right)=L_{i}\left(t\right)\times A\times \left(1-K_{d}\right)\times f_{\text{wash}}\;\text{.} \end{array}$$

Here, $W_{i}\left(t\right)$ (in CFU/d) is the total bacterial load washed off from the field on day t in simulation i. $L_{i}\left(t\right)$ (in CFU/m^2^) is the soil-module output, A (in m^2^) is the field area, $K_{d}$ is the partition coefficient (fraction of bacteria sorbed to soil), and $f_{\text{wash}}$ is the wash-off fraction of freely mobile bacteria. We compute wash-off once per day as a fixed fraction of the field load that is free for transport, representing the expected (event-averaged) daily export due to rainfall/runoff. By routing $W_{i}\left(t\right)$ directly into the river on the same day, we are effectively assuming that the farm-to-stream distance is short enough that travel time can be ignored at daily resolution.

#### 2.4.2 In-stream decay rate

Mancini's Equation [44] was applied to capture in-river *E. coli* decay as a function of water temperature (T), salinity, and solar radiation. The daily first-order decay rate *k* was described as:

(18) $$\begin{array}{c} k=0.8+0.006\times EC\times 1.07^{\left(T-20\right)}+\frac{\text{IA}\times \left[1-\exp\left(-e\times H\right)\right]}{e\times H}\text{,} \end{array}$$

where EC is salinity (in %), IA is the solar radiation (in ly/h), e is the light extinction coefficient, and H is water column depth (in m).

#### 2.4.3 Accumulated river load

(19) $$\begin{array}{c} M_{i}\left(t\right)=M_{i}\left(t-1\right)\;\exp\left(-k\right)+W_{i}\left(t\right),M_{i}\left(0\right)=W_{i}\left(0\right)\text{,} \end{array}$$

where $M_{i}\left(t\right)$ (in CFU) is the cumulative ESBL *E. coli* count in the water site on day t. Each day, the previous day's count decays by exp⁡(−k) and the new wash-off input $W_{i}\left(t\right)$ is added.

Dividing the accumulated $M_{i}\left(t\right)$ by the site volume V (in L) yields *the* ESBL *E. coli* concentration $C_{i}\left(t\right)$ in CFU/L:

(20) $$\begin{array}{c} C_{i}\left(t\right)=\frac{M_{i}\left(t\right)}{V}\text{.} \end{array}$$

**Time notation (soil and river modules).** We define continuous time t as days since litter application, with t=0 at application. Simulation outputs are stored and reported on discrete post-application days d=1,2,…, where day d represents the interval t∈(d−1,d]. This notation represents processes updated on a daily (24-hour) time step after application, while *t* = 0 is reserved as the instantaneous initial boundary condition. The soil module outputs loads d≥1, the river module uses the same day index with zero initial load at t=0 and updates concentrations from the day d export and in-stream decay.

### 2.5 Lettuce module

To estimate *E. coli* contamination on lettuce, we linked daily irrigation-water concentrations $C_{i}\left(t\right)$ (in CFU/mL) from the river model to leaf-surface adhesion and biphasic die-off on the crop (Table 3), following a previously proposed framework by O'Flaherty [19]. We evaluated scenarios with different intervals between land application of litter and lettuce planting. We define ΔAP as the days between application of litter and planting, with the environmental time origin at t=0 on the day of application, thus planting occurs at t=ΔAP. In the lettuce module, irrigation begins on the planting day and proceeds daily through the growth period (35 d), so ΔAP is operationally equivalent to the interval from application to the first irrigation event. Leaf die-off is indexed on a leaf clock τ=1,2,…,35.

**Table 3:** Key input variables for the lettuce module.

Variable	Description	Unit	Value	Source
ΔAP	Days between litter application and lettuce planting	d	1, 20, 50, 100, 150	User defined
f	Fast-phase fraction in biphasic decay	Fraction	7.6 × 10^−5^	[19]
$k_{1}$	Fast decay coefficient	d	4.45	[19]
$k_{2}$	Slow decay coefficient	d	0.0698	[19]
$V_{\text{attach},i}$	Leaf-film water volume	mL/g	0.006 + LN (−4.75, 0.50)	[19]
$D_{\text{treat}}$	Producer-level washing reduction factor	Fraction	0.826	[19]
$D_{\text{wash},i}$	Consumer washing efficiency	Fraction	Triangular 0.65, 0.99, 0.99	[19]
$S_{portion}$	Portion size for risk estimate	g	100	[19]
T	Days from planting to harvest	d	35	User defined
$r_{\text{DR}}$	Exponential dose–response parameter	CFU	2.18 × 10^−6^	[45]
$F_{\text{UPEC}}$	Fraction of *Escherichia coli* that are uropathogenic	Fraction	0.1	[46]
$p_{\text{colon}|\text{gut}}$	P (urinary colonization)	Probability	Uniform 0.35, 0.46	[46]
$p_{\text{UTI}|\text{colon}}$	P (symptomatic UTI|urinary colonization)	Probability	0.067	[46]
δ	DALY burden per UTI case	DALY/case	Uniform 3.70, 12.84	[46]

Abbreviations: UTI, urinary tract infection; DALY, disability-adjusted life year; CFU, colony-forming unit.

#### 2.5.1 Leaf-surface adhesion

(21) $$\begin{array}{c} E_{\text{adh},i}\left(t\right)=C_{i}\left(t\right)\times V_{\text{attach},i}\text{,} \end{array}$$

where $E_{\text{adh},i}\left(t\right)$ (in CFU/g) is the bacteria adhering to leaves, and $V_{\text{attach},i}$ (in mL/g) is the water-attachment volume.

#### 2.5.2 Biphasic decay on leaves

(22) $$\begin{array}{c} {l\text{og}}_{10}\;\left[E_{\text{obs},i}\left(t\right)\right]=f\;\left(-k_{1}\;t\right)+\left(1-f\right)\;\left(-k_{2}\;t\right)\text{,} \end{array}$$

and

(23) $$\begin{array}{c} E_{\text{decay},i}\left(t\right)=E_{\text{adh},i}\left(t\right)\times 10^{{l\text{og}}_{10}\left[E_{\text{obs},i}\left(t\right)\right]}\text{,} \end{array}$$

where a fraction f of cells decays at the fast rate $k_{1}$ (in d^−1^) and the remainder (1−f) at the slower rate $k_{2}$ (in d^−1^). Here, t is days since first irrigation after planting.

#### 2.5.3 Accumulated contamination at harvest

(24) $$\begin{array}{c} E_{\text{cum},i}=\sum_{t=p}^{p+T-1}E_{\text{adh},i}\left(t\right)-\sum_{t=p}^{p+T-1}E_{\text{decay},i}\left(t\right)\text{,} \end{array}$$

where p is the planting day, T is the growth duration (35 d), and the sums collect adhesion and decay over the growth period to compile the accumulated contamination at harvest $E_{\text{cum},i}$.

#### 2.5.4 Post-harvest washing

A washing reduction factor $D_{\text{treat}}$ represents the fraction of bacteria removed by producer washing with water. The concentration at harvest is calculated as:

(25) $$\begin{array}{c} E_{\text{post},i}=E_{\text{cum},i}\times D_{\text{treat}}\;\text{.} \end{array}$$

Furthermore, we modelled two scenarios: the consumption of 100 g of lettuce $S_{portion}$ of unwashed lettuce, as:

(26) $$\begin{array}{c} E_{\text{unwashed},i}=S_{portion}\times E_{\text{post},i}\text{,} \end{array}$$

and the consumption of $S_{portion}$ with an additional home washing ($D_{\text{wash},i})$, as:

(27) $$\begin{array}{c} E_{\text{final},i}^{100g}={S_{portion}\times E}_{\text{post},i}\times \left(1-D_{\text{wash},i}\right) \end{array}.$$

#### 2.5.5 Probability of infection and DALY estimation

We mapped lettuce dose (CFU per 100 g serving) to health outcomes using a pooled exponential dose–response for enteropathogenic *E. coli* (EPEC) with a fecal-shedding endpoint as a surrogate for gastrointestinal (GI) colonization. The parameterization ultimately traces to human volunteer challenge with EPEC O127:H6 (strain E2348/69); colonization in those studies was determined by recovery/shedding of the challenge strain from stools (primary source and trial details in Tacket et al. [45]). We adopted this surrogate because ESBL- or uropathogenic *E. coli* (UPEC)-specific human DR data are not available, using the approach taken by Heida et al. [46] for ESBL *E. coli* in recreational water.

To translate GI colonization to urinary tract infection (UTI) burden, we followed Heida's framework [46]: (1) apply a UPEC-like fraction to the ingested ESBL population; (2) use conditional probabilities for urinary tract colonization (given gut colonization) and for symptomatic UTI (given urinary colonization) as independent transition probabilities; and (3) compute DALY per serving from outcome probabilities and published disability weights/durations.

The probability of GI is calculated as:

(28) $$\begin{array}{c} P_{\text{gut}}=1-\exp\left(-r_{\text{DR}}E_{k}F_{\text{UPEC}}\right)\text{,} \end{array}$$

where $r_{\text{DR}}$ is the exponential dose–response parameter, $E_{k}$ is the ingested dose (in CFU) in a single event (lettuce consumption) and $F_{\text{UPEC}}$ is the fraction of ESBL *E. coli* that are uropathogenic.

Chicken feces (and thus litter) commonly contain *E. coli* populations in which a minority meet extraintestinal pathogenic *E*. *coli* (ExPEC) criteria, and some of these are UPEC-like; published prevalences range from ∼13 % ExPEC in broiler feces (with many categorized as UPEC) to ∼23 % ExPEC in retail poultry meat. We therefore set $F_{\text{UPEC}}$ = 0.10 as a realistic, conservative central, in accordance with Heida [[46], [47], [48], [49], [50]].

The probability of UTI is estimated as:

(29) $$\begin{array}{c} P_{\text{UTI}}=P_{\text{gut}}\times p_{\text{colon}|\text{gut}}\times p_{\text{UTI}|\text{colon}}\text{,} \end{array}$$

where $p_{\text{colon}|\text{gut}}$ is the conditional probability of urinary tract colonization (given gut colonization) and $p_{\text{UTI}|\text{colon}}$ is the conditional probability of symptomatic UTI (given urinary tract colonization).

Finally, we estimated the DALY as:

(30) $$\begin{array}{c} \text{DALY}=P_{\text{UTI}}\times \delta \text{,} \end{array}$$

where δ is the DALY burden per UTI case (DALY/case).

### 2.6 Sensitivity analysis

We performed a variance-based sensitivity analysis using Partial Rank Correlation Coefficients (PRCC) to quantify how variability in the model inputs propagates to uncertainty in the litter load and in the final exposure outputs.

First, we identified key parameters spanning from each module, each varied uniformly over ±50 % of its baseline value. We generated 1000 stratified Latin Hypercube Sampling (LHS) draws across the *n*-dimensional input space, ensuring full coverage of each parameter's marginal distribution without clustering.

For each of the 1000 parameter vectors, we executed the complete simulation chain, extracting the mean human exposure. Runs were parallelized with independent L'Ecuyer-Combined Multiple Recursive Generator substreams [51].

Once the vector of output metrics was assembled, we computed PRCCs and derived 95 % confidence intervals (*CI*s) through 100 bootstrap replicates. PRCC measures the monotonic relationship between each input and the outcome, controlling for all other parameters; those whose intervals excluded zero were deemed statistically significant.

### 2.7 Software

All analyses and simulations were implemented in R (version 4.3.1) and RStudio (version 2024.09.1 build 394). Key packages including “tidyverse” [52] for data manipulation and visualization, “mc2d” [53] and “triangle” [54] for distribution sampling, “nls2” [55] for nonlinear regression, “furrr” [56] for parallel computation, “sensitivity” [57], and “lhs” [58] for sensitivity analysis. The full QMRA model is openly available on ENVIRE GitHub (https://github.com/ENVIRE-JPIAMR).

## 3 Results and discussion

### 3.1 Farm module

**Fig. 3:**
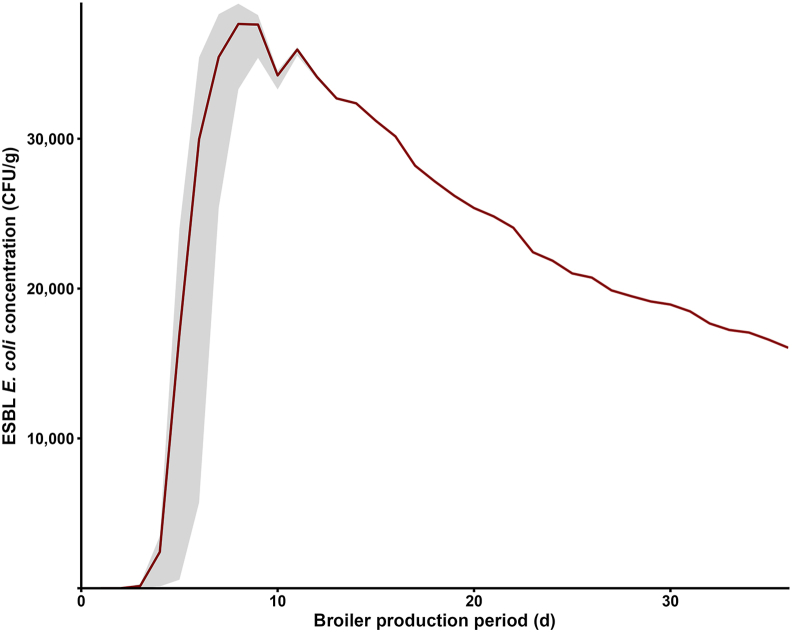
ESBL *Escherichia coli* concentration (CFU/g) in litter over the broiler production period. Mean (dark red line) with 95 % uncertainty interval (shaded grey; 2.5th–97.5th percentiles) of 1000 iterations. Abbreviations: ESBL, extended-spectrum β-lactamase; CFU, colony-forming unit.

ESBL *E. coli* concentration in litter rose sharply after day 2 (16.70 × 10^−3^ CFU/g, SD: 5.00 × 10^−4^, 95% *UI*: 7.66 × 10^−4^–2.70 × 10^−^^3^), reaching a maximum at day 8 (3.76 × 10^4^ CFU/g, SD: 1.73 × 10^3^, 95% *UI*: 3.33 × 10^4^–3.90 × 10^4^), before declining to 1.60 × 10^4^ CFU/g (SD: 16.07, 95% *UI*: 1.60 × 10^4^–1.60 × 10^4^) at day 36 (Fig. 3).

**Fig. 4:**
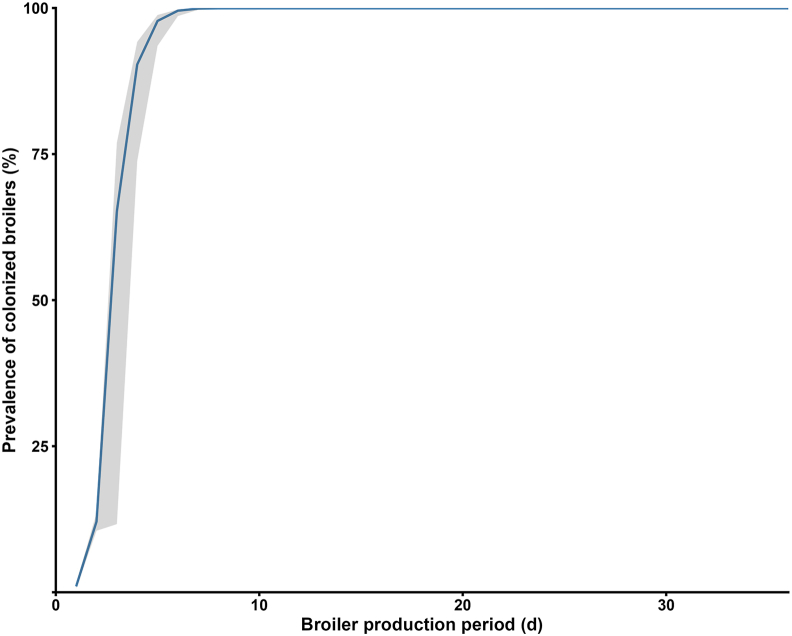
Prevalence of colonized broilers over time. Mean (blue line) with 95 % uncertainty interval (shaded grey; 2.5th–97.5th percentiles) of 1000 iterations.

The proportion of colonized broilers climbed from 65.2 % (95% *UI*: 11.6 %–76.9 %) on day 3 to more than 99.9 % by day 7 (95% *UI*: 99.7 %–99.9 %), remaining essentially constant thereafter (Fig. 4).

The farm module simulates a rapid and efficient spread of ESBL *E. coli* within broiler flocks, consistent with field data and experimental studies [[59], [60], [61], [62]]. In our simulation, flock-level colonization approached 100 % within the first week. This pattern is similar to longitudinal studies where prevalence in day-old chicks often rises from negligible levels to near saturation within 5–7 d [6,60,61]. For example, Kasparaviciene et al. [60] detected no ESBL *E. coli* on day 0, but found a 57.5 % prevalence by day 5, while Huijbers et al. [61] reported increases from 0 % to 24 % at day 1 to over 96 % by day 7. These dynamics are biologically plausible given the extremely low infectious dose required for colonization, as few as 10–100 CFU can establish persistent colonization in broilers [12,62].

The concentration of ESBL *E. coli* in the litter estimated by this module aligns with the lower to mid-range of reported concentrations, though literature values vary widely [63]. measured ESBL/AmpC-*E. coli* loads in broiler cecal contents between 2.85 and 4.17 log_10_ (CFU/g), while Atanasova et al. [27] reported up to 5.48 log_10_ (CFU/g) in fresh broiler litter. At the lower end, Blaak et al. [64] found a mean concentration of 2.4 × 10^4^ CFU/kg in feces from Dutch farms. Also, Becker et al. [39] quantified a lower number of CFU/g in broiler litter. Such discrepancies are likely influenced by multiple factors including broiler age, diet, specific bacterial strains, litter conditions, sampling methods, and farm management. The model's simplified assumption of logistic growth and a constant excretion rate does not fully capture transient increases in shedding that may occur due to stressors such as thinning, transport, or diet. As a result, the model may underestimate peak shedding under certain real-world scenarios. Finally, our model underlines a critical message: once ESBL-producing *E. coli* is introduced in the barn, it spreads rapidly and is difficult to suppress [10,65]. Because broiler colonization requires only a minimal infectious dose, even small biosecurity lapses at chick placement can result in widespread contamination.

In our simulation, we applied a Susceptible-Infected (colonized) (SI) transmission model, assuming that once a broiler becomes colonized it remains so until the end of the production cycle. Persistent colonization of gut bacteria like *E. coli* in broilers is well documented [12], yet this simplification may overlook more complex within-host dynamics [66]. Dankittipong et al. [67] compared both SI and Susceptible-Infectious-Susceptible (SIS) frameworks to study carbapenemase- and ESBL-producing *E. coli*, highlighting different transmission rates among resistant strains. Likewise, Becker et al. [39] and Dame-Korevaar et al. [68] found the SI model is adequate for capturing within-flock spread in broilers. In contrast, Furusawa et al. [69] developed two compartmental models—one with a time-dependent decline in susceptibility and one with partial immunity to phylogenetic groups—to describe ESBL-producing *E. coli* transmission in Dutch broiler chains. Those models also incorporated environmental contamination between production cycles and within flocks, offering a more detailed framework for testing interventions and estimating public health risk. Nevertheless, given the evidence for long-term carriage in broiler chickens, we judged the SI model the most consistent with our “worst case scenario” approach, since it reflects a permanent infection state once colonization occurs.

**Fig. 5:**
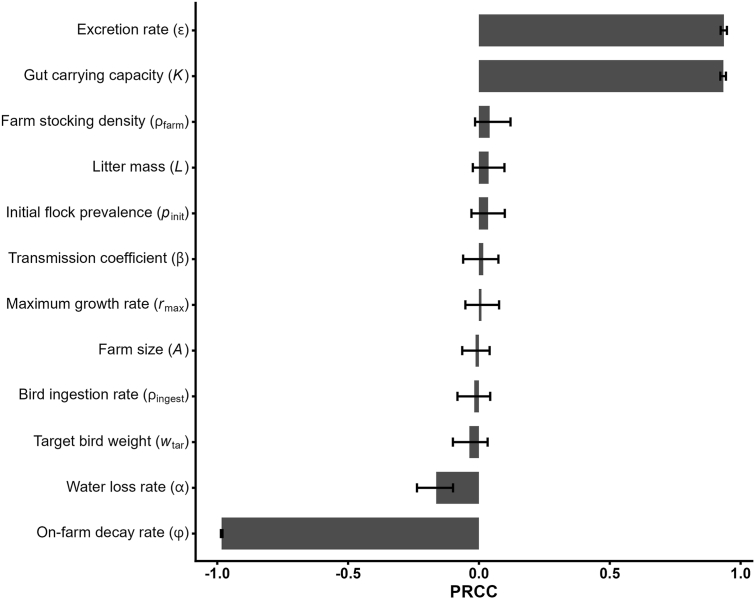
Sensitivity analysis of litter ESBL *Escherichia coli* load (CFU/g litter) at day 36; displayed is the mean PRCC with 95 % confidence intervals. Abbreviations: CFU, colony-forming unit; PRCC, Partial Rank Correlation Coefficient.

The sensitivity analysis of the farm module highlights three overwhelmingly dominant drivers of within-flock bacterial loads (Fig. 5). First, the on-farm decay rate ϕ shows a very strong negative correlation with mean litter *E. coli* concentration (PRCC ≈ −0.98), indicating that small increases in bacterial die-off sharply reduce end-cycle loads. Conversely, gut carrying capacity K (PRCC ≈ +0.93) and the excretion rate *ε* (PRCC ≈ +0.93) both exhibited very strong positive correlations, underscoring that higher maximal gut loads and greater per-bacterium excretion rates drive litter contamination. Secondary factors include water metabolic loss α (PRCC ≈ −0.16) and target broiler weight $w_{\text{tar}}$ (PRCC ≈ −0.04), which have minor negative effects. All other parameters have PRCCs near zero with 95% *CI*s spanning zero, indicating negligible impact under the studied ranges. These results point to the critical importance of both bacterial die-off dynamics and host-level factors (gut capacity and shedding) in shaping ESBL *E. coli* loads on broiler farms.

### 3.2 Soil and river modules

When expressed per m^2^, the mean ESBL *E. coli* loads started at 3.2 × 10^7^ (SD: 3.2 × 10^4^, 95% *UI*: 3.2 × 10^7^–3.2 × 10^7^) CFU/m^2^ on day 1 post litter application, fell to 2.2 × 10^7^ (SD: 2.2 × 10^4^, 95% *UI*: 2.2 × 10^7^–2.2 × 10^7^) CFU/m^2^ at day 10, and declined further to 8.6 × 10^5^ (SD: 8.8 × 10^2^, 95% *UI*: 8.6 × 10^5^–8.6 × 10^5^) CFU/m^2^ by day 100. This per-area view mirrors the per-mass decay and confirms that fields receive a substantial bacterial pulse immediately after litter application, but surface-area concentrations drop substantially within three months. Consequently, while broiler litter is a pronounced short-term source of soil contamination, its long-term contribution to human exposure via soil contact is minimal under baseline conditions. A limitation of our model is the lack of studies to use for validating the soil module. Most of the literature focuses on generic *E. coli* [32] or uses of soil (in CFU/g) mixed with manure as quantitative output [70]. It is also important to specify that another big limitation of our model is the simplified litter application, as we assumed the ESBL *E. coli* to spread uniformly following a fixed application rate.

Simulated ESBL *E*. *coli* in river water decreased from 8.3 × 10^−2^ CFU/mL (SD: 4.8 × 10^−3^, 95% *UI*: 7.6 × 10^−2^–9.2 × 10^−2^) on day 1 to 6.0 × 10^−2^ CFU/mL (SD: 3.5 × 10^−3^, 95% *UI*: 5.5 × 10^−2^–6.6 × 10^−2^) by day 10 and reached 6.2 × 10^−5^ CFU/mL (SD: 3.6 × 10^−6^, 95% *UI*: 5.7 × 10^−5^–6.9 × 10^−5^) by day 200. Early declines were modest, reflecting dilution and decay; by day 200, concentrations were essentially zero.

These magnitudes fall within reported bathing-water ranges for antibiotic resistant/ESBL *E. coli* (predicted 0.45–345.09 CFU/100 mL) as summarized by O'Flaherty et al. [18] and are consistent with ESBL fractions (0.05 %–1.00 %) applied to recreational criteria (0.06–4.10 CFU/100 mL) in Heida et al. [46].

### 3.3 Lettuce module

**Fig. 6:**
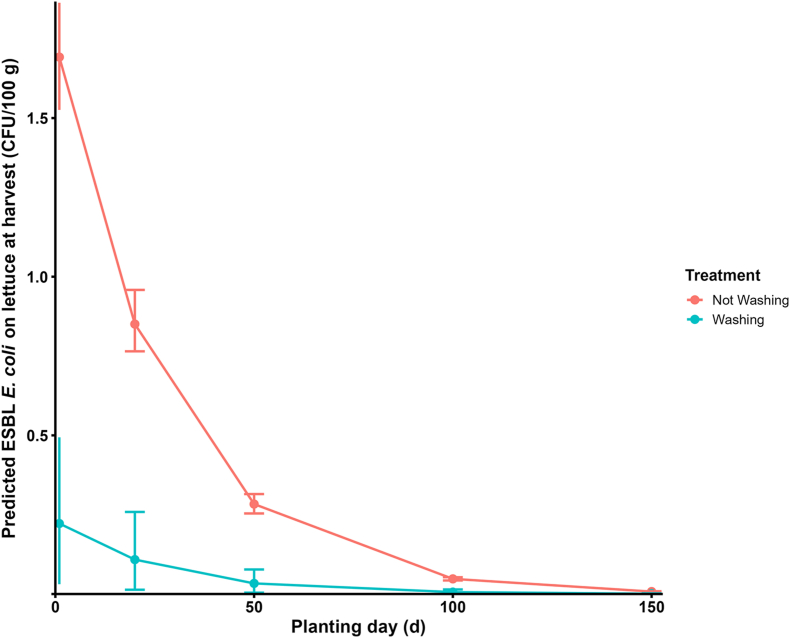
Predicted ESBL *Escherichia coli* on lettuce at harvest (CFU/100 g) at different discrete intervals between broiler litter application and planting day. Lines and points show mean exposure; error bars show the 95 % uncertainty interval (2.5th–97.5th percentiles) across 1000 iterations. Abbreviations: ESBL, extended-spectrum β-lactamase; CFU, colony-forming unit.

Our model simulated the contamination of lettuce via irrigation with water containing ESBL *E. coli*, predicting contamination levels at harvest influenced by river contamination, application practices, and post-irrigation environmental factors, with risk reduction achieved through standard household washing. In our simulation, we estimated the exposure via ingestion of both consumer-washed and unwashed lettuce. Furthermore, we compared harvest-time exposure across various intervals between litter application and lettuce planting. With a one-day interval, mean exposure reached 1.70 CFU/100 g (SD: 8.58 × 10^−2^, 95% *UI*: 1.52–1.86) for unwashed lettuce and 0.22 CFU/100 g (SD: 0.13, 95% *UI*: 0.03–0.49) after a consumer wash, indicating that even simple rinsing may reduce loads by nearly 90 % (Fig. 6). As the interval lengthened, exposures fell markedly: at 20 d, 0.85 CFU/100g (SD: 5.01 × 10^−2^, 95% *UI*: 0.76–0.95) (unwashed) and 0.10 CFU/100g (SD: 7.42 × 10^−2^, 95% *UI*: 0.01–0.25) (washed); at 150 days, 7.66 × 10^−3^ CFU/100g (SD: 4.50 × 10^−4^, 95% *UI*: 6.75 × 10^−3^–8.60 × 10^−3^) and 8.64 × 10^−4^ CFU/100g (SD: 5.57 × 10^−4^, 95% *UI*: 1.12 × 10^−4^–2.03 × 10^−4^), respectively.

Under our scenarios (ΔAP = 1–150 d; washed vs. unwashed), UTI risk per serving ranges from 9.0 × 10^−9^ at ΔAP = 1 d (unwashed) to 4.6 × 10^−12^ at ΔAP = 150 d (washed), i.e., about one case per 1.1 × 10^8^ to 2.2 × 10^11^ servings. DALY per serving ranges from 7.5 × 10^−8^ to 3.7 × 10^−11^, i.e., roughly 1 DALY per 1.3 × 10^7^ to 2.7 × 10^10^ servings. For completeness, GI colonization risk spans 3.3 × 10^−7^ to 1.7 × 10^−10^, equivalent to 1 per 3.0 × 10^6^ to 5.9 × 10^9^ servings. Extending the planting interval from ΔAP = 1 to ΔAP = 150 days reduces UTI and DALY by 2.3–2.4 orders of magnitude, while household washing adds a further reduction at any ΔAP. Combined, ΔAP + washing lowers UTI and DALY by ≈ 3.3 orders of magnitude. These are scenario-specific indicators as there is no accepted DALY per serving benchmark in foods, and they align with expected mechanisms of pre-irrigation die-off and reduced bacteria load with washing (Table 4). Consumer washing and increasing the interval between litter application and lettuce planting reduced colonization risks, UTI risks, and DALYs. It appears that the rapid natural decay of *E. coli* on foliage and the incremental benefit of simple post-harvest washing reduce the risk of UTI and DALY burden from raw-leaf consumption. Our results are consistent with studies identifying irrigation water as a plausible route for pre-harvest contamination of fresh produce. However, we did not model or compare alternative routes and therefore do not rank their relative importance [15,[71], [72], [73]]. Several studies have shown that *E. coli*, including pathogenic strains, can attach to and survive on lettuce surfaces for extended periods [74,75]. Environmental factors such as higher temperatures and light intensity tend to speed inactivation on plant surfaces [76,77]. In our model, increasing the interval between litter application and planting reduces predicted contamination. This is consistent with studies showing time-dependent decline of *E. coli* in manure-amended soils and reduced detection on crops with longer intervals after application [78,79]. Across all scenarios, exposure at harvest decreased with increasing the interval between manure application and planting. Mechanistically, the interval increases the window for soil die-off before irrigation begins, which aligns with the sensitivity finding that soil decay is one of the main exposure drivers. From a management perspective, the interval between manure application and planting can be considered alongside irrigation practices and manure handling as part of a first line strategy to reduce the exposure.

**Table 4:** Mean and 95% *UI* (in brackets) probability of GI colonization and UTI, and corresponding DALY per exposure event for each planting interval and exposure scenario (washed vs. unwashed lettuce).

Planting interval	Risk of GI	Risk of UTI	DALY	Exposure
1	3.3 × 10^−7^ (2.9 × 10^−7^–3.6 × 10^−7^)	9.0 × 10^−9^ (7.5 × 10^−9^–1.0 × 10^−8^)	7.5 × 10^−8^ (3.4 × 10^−8^–1.2 × 10^−7^)	Unwashed
1	4.3 × 10^−8^ (6.0 × 10^−9^–9.6 × 10^−8^)	1.1 × 10^−9^ (1.6 × 10^−10^–2.6 × 10^−9^)	1.0 × 10^−8^ (1.2 × 10^−9^–3.0 × 10^−8^)	Washed
20	1.6 × 10^−7^ (1.5 × 10^−7^–1.8 × 10^−7^)	4.5 × 10^−9^ (3.6 × 10^−9^–5.2 × 10^−9^)	3.6 × 10^−8^ (1.7 × 10^−8^–5.8 × 10^−8^)	Unwashed
20	2.1 × 10^−8^ (2.6 × 10^−9^–5.0 × 10^−8^)	5.8 × 10^−10^ (7.1 × 10^−11^–1.3 × 10^−9^)	4.7 × 10^−9^ (4.9 × 10^−10^–1.3 × 10^−8^)	Washed
50	5.5 × 10^−8^ (4.9 × 10^−8^–6.1 × 10^−8^)	1.5 × 10^−9^ (1.2 × 10^−9^–1.8 × 10^−9^)	1.1 × 10^−8^ (5.6 × 10^−9^–1.8 × 10^−8^)	Unwashed
50	6.5 × 10^−9^ (8.2 × 10^−10^–1.5 × 10^−8^)	1.7 × 10^−10^ (2.2 × 10^−11^–4.3 × 10^−10^)	1.5 × 10^−9^ (1.6 × 10^−10^–3.9 × 10^−9^)	Washed
100	9.3 × 10^−9^ (8.3 × 10^−9^–1.0 × 10^−8^)	2.5 × 10^−10^ (2.1 × 10^−10^–3.0 × 10^−10^)	2.2 × 10^−9^ (9.0 × 10^−10^–3.4 × 10^−9^)	Unwashed
100	1.1 × 10^−9^ (1.5 × 10^−10^–2.8 × 10^−9^)	3.1 × 10^−11^ (3.6 × 10^−12^–7.8 × 10^−11^)	2.3 × 10^−10^ (2.2 × 10^−11^–5.4 × 10^−10^)	Washed
150	1.5 × 10^−9^ (1.3 × 10^−9^–1.6 × 10^−9^)	4.0 × 10^−11^ (3.4 × 10^−11^–4.9 × 10^−11)^	3.2 × 10^−10^ (1.5 × 10^−10^–5.1 × 10^−10^)	Unwashed
150	1.7 × 10^−10^ (2.2 × 10^−11^–3.9 × 10^−10^)	4.6 × 10^−12^ (5.6 × 10^−13^–1.0 × 10^−11^)	3.7 × 10^−11^ (4.2 × 10^−12^–1.0 × 10^−10^)	Washed

Abbreviations: *UI*, uncertainty interval; GI, gastrointestinal; UTI, urinary tract infection; DALY, disability adjusted life year.

Regarding post-harvest mitigation at the consumer level, the model prediction of limited efficacy on *E. coli* complete reduction for household washing is strongly supported by experimental data [[80], [81], [82]]. However, properly rinsing green leaves may reduce the consumer's risk [77]. The limitations of consumer-level washing practices firmly place the primary responsibility for ensuring the microbial safety of fresh produce, particularly regarding contaminants introduced via irrigation, on upstream controls within the agricultural production system.

**Fig. 7:**
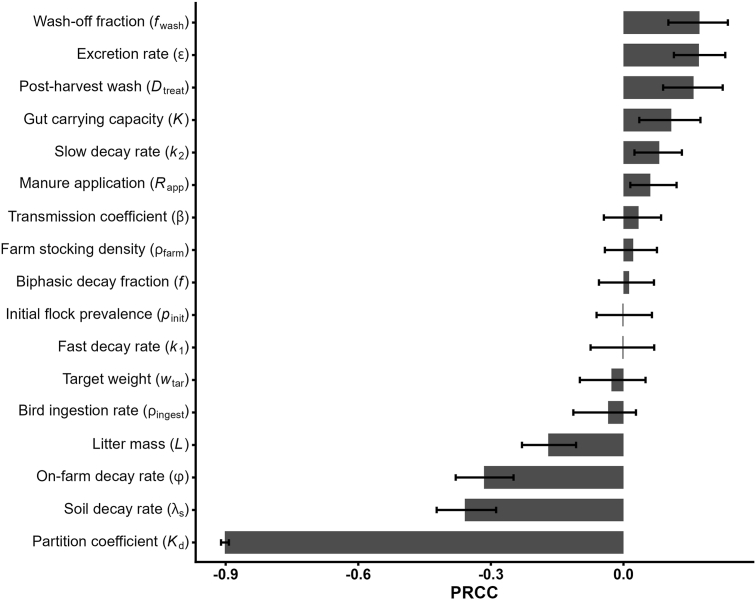
Sensitivity analysis of lettuce consumption (single event). Displayed is the mean PRCC with 95 % confidence intervals. Abbreviation: PRCC, Partial Rank Correlation Coefficient.

The sensitivity analysis (Fig. 7) showed that lettuce-borne ESBL *E. coli* exposure is overwhelmingly governed by how readily bacteria sorb to soil particles: the partition coefficient has a very strong negative association (PRCC ≈ −0.90), meaning that greater sorption reduces transfer to runoff and, ultimately, to foliage [14]. Soil decay kinetics also matter: the estimated decay rate in soil and the environmental decay rate on the farm both correlate negatively (PRCC ≈ −0.36 and −0.31, respectively), indicating that faster bacteria die-off sharply cuts lettuce contamination. On the flip side, parameters that increase bacterial availability such as wash-off fraction from soil to water (PRCC ≈ +0.17) and the shedding rate in the farm module (PRCC ≈ +0.17) modestly raise exposure. Litter mass (in kg/m^2^) and gut carrying capacity also exert smaller but significant influences. Together, these results translate into practical levers and measurement priorities: the strong influence of soil-water partitioning supports targeted field tests to quantify the locally particle-associated fraction; the effects of soil and on-farm decay rates point to manure treatment and crop scheduling that maximize pre-irrigation die-off; positive influence of runoff parameters argues for runoff management (e.g., buffers, cover structure, and irrigation timing) to limit mobilization; flock shedding effects reinforce on-farm colonization controls (competitive exclusion vaccines, for example). Finally, the planting interval functions as a simple scheduling knob that works additively with consumer washing to reduce dose. Accordingly, the highest-value measurements to reduce uncertainty are site-specific soil-water partition coefficients, soil die-off under local temperature and moisture regimes, and plot-scale runoff export.

## 4 Discussion

Our model incorporates several simplified assumptions that merit further refinement. In general, our model represents a worst-case scenario, potentially overestimating human exposure. First, in the farm module, we treat *E. coli* as uniformly distributed across all fecal matter and assume each broiler remains colonized from the moment of infection until cycle end, with bacterial growth governed by a single logistic curve. In reality, fecal deposition is spatially clustered, broiler immune responses and stress levels vary over time, and diversity among ESBL *E. coli* strains can differ, factors that can drive transient “hot spots” of shedding and alter transmission dynamics.

Second, the soil module idealizes litter spreading as perfectly homogeneous and represents die-off with one constant exponential rate. This ignores the influence of temperature fluctuations, soil moisture, pH gradients, bioturbation by earthworms and other fauna, and incorporation by tillage all of which can create microsites where bacteria persist or die off more rapidly. Furthermore, litter is assumed to be spread on fields immediately after flock removal (0 days storage) and without any treatment (such as composting), ensuring maximal viable ESBL *E. coli* loads at application. Nevertheless, although manure treatment and storage are common practices [32], the Nitrates Directive (91/676/EEC), the main EU legislation regulating manure use to protect water quality, does not mandate treatments or set a waiting period.

Third, our river transport component applies fixed wash-off fractions and partition coefficients, yielding a smooth decline in contaminant load. Yet real watersheds undergo storm-driven pulses, variable flow regimes, sediment resuspension, channel morphology effects, and spatial heterogeneity in land cover, which together can produce episodic peaks in bacterial concentrations that our model may miss. As a potential improvement, incorporation of a full SWAT model could better capture these complex hydrological and land-use dynamics [38].

Fourth, the lettuce module relies on a biphasic decay curve with uniform attachment efficiency to leaf surfaces and a single removal efficiency for post-harvest washing. It does not account for differences in leaf microstructure, irrigation method (e.g., overhead versus drip), spray droplet size, canopy microclimate, or commercial-scale wash protocols.

Furthermore, our framework omits key microbial ecology processes, most notably horizontal transfer of resistance genes (e.g. via plasmids) among environmental bacteria and interactions with other microbial populations that may inhibit or promote ESBL *E. coli* survival. We also do not capture potential regrowth phases under favorable conditions.

Another dominant uncertainty is the soil-water attachment/partitioning of ESBL *E. coli* under local conditions—i.e., the proportion that is particle-associated vs. freely suspended. Ideal data would quantify, after rainfall or irrigation in litter-amended plots, bacteria in the water phase and in the sediment phase, or use simple laboratory tests to estimate the particle-association fraction for local soils.

Our dose–response model maps from an EPEC human challenge model (fecal shedding as a proxy for GI colonization) to UTI via conditional transitions and a UPEC-like fraction. This surrogate is necessary because no ESBL- or UPEC-specific human dose–response exists; consequently, risk outputs should be interpreted as scenario-based indicators with uncertainty concentrated in the UPEC fraction and conditional transitions rather than as clinical predictions. Heida et al. [46] emphasized the same caveats for recreational waters; we adopt that caution here. Evidence to date documents contamination of raw vegetables with ESBL/ExPEC *E. coli* but direct epidemiologic links between raw-vegetable consumption and UTI are limited [83,84]. The biological plausibility rests on GI colonization serving as a reservoir for UPEC and periurethral transfer, with sex differences in UTI incidence not resolved in our per-serving DALY [85].

Finally, to our knowledge, this is the first ESBL *E. coli* focused QMRA connecting broiler flock, litter-amended soil, receiving water and lettuce consumption in a single framework. For this reason, a head-to-head validation against a prior end-to-end model is not yet possible. We therefore adopted a modular validation strategy and, where feasible, compared predicted magnitudes and expected signatures with published models and experimental studies.

Moreover, broiler-derived ESBL *E. coli* datasets are not available for the soil, river, and lettuce stages. Available studies typically report indicator *E. coli* or ESBL from mixed sources or use non-comparable units, which precludes a like-for-like error metric (e.g., Root Mean Squared Error/bias) for broiler-attributed ESBL. To avoid over-interpreting proxy data as benchmarks, we therefore limited validation to modular plausibility checks and report parameter influence via global sensitivity analysis.

Despite these simplifications, the model captures the dominant processes governing ESBL *E. coli* dissemination to produce and, by intentionally adopting conservative assumptions, provides exposure estimates that likely bound real-world outcomes. Consequently, we believe the results remain sufficiently robust to inform risk management and guide intervention strategies.

Future model iterations should introduce spatially explicit litter application, temperature and moisture driven decay rates, event-based hydrologic inputs, mechanistic leaf-surface colonization dynamics, and microbial community interactions, including gene transfer dynamics. Incorporating these elements will enhance realism, reduce uncertainty, and yield more robust estimates of human exposure risk.

## 5 Conclusions

In this study, we developed and applied a four-module QMRA model to trace the journey of ESBL *E. coli* from broiler flocks through soil, surface water, and fresh produce. Beyond quantifying exposure, the primary contribution of this work is a transferable modelling framework that integrates broiler production, environmental transmission, and food exposure, making the chain of assumptions explicit and enabling systematic comparison of mitigation options. Sensitivity analyses identified the soil-water partition coefficient and decay rates as the most influential parameters, pointing to targeted data collection needs. Although natural decay, dilution, and interventions such as composting can reduce human exposure by orders of magnitude, persistent contamination calls for robust manure management and strict irrigation-water quality controls. This work reinforces the importance of aligning manure management practices with irrigation-water quality safeguards, because environmental pathways can link agricultural decisions to downstream human exposure. The model also clarifies which parameters dominate uncertainty, providing a clear agenda for targeted data collection and monitoring that would most improve confidence in future assessments. Future work should introduce spatial heterogeneity in litter spreading, dynamic weather drivers, host immunity and horizontal gene transfer to refine risk estimates and guide effective on-farm and pre-harvest interventions.

## CRediT authorship contribution statement

**Nunzio Sarnino:** Writing – review & editing, Writing – original draft, Visualization, Software, Methodology, Formal analysis, Data curation, Conceptualization. **Subhasish Basak:** Writing – review & editing, Software, Methodology, Formal analysis, Conceptualization. **Lucie Collineau:** Writing – review & editing, Validation, Supervision, Project administration. **Roswitha Merle:** Writing – review & editing, Validation, Supervision, Project administration, Funding acquisition.

## Funding

This work is a part of the European project ENVIRE funded by the JPIAMR program of the European Union, and funded by the German Federal Ministry for Research and Education (Support Code: 01KI2202A).

## Declaration of competing interest

The authors declare that they have no known competing financial interests or personal relationships that could have appeared to influence the work reported in this paper.
